# An audit of postoperative haemodynamic stability after intraoperative labetalol administration in non-cardiac surgery patients

**DOI:** 10.1177/17504589231223011

**Published:** 2024-02-11

**Authors:** Benjamin B Cairns, Megan V McNeil, Andrew D Milne

**Affiliations:** Dalhousie University, Halifax, NS, Canada

**Keywords:** Labetalol, Antihypertensive agents, Therapeutic use, Haemodynamics, Drug effects, Postoperative complications, Drug therapy

## Abstract

Anaesthesiologists commonly use intravenous labetalol to adjust patient haemodynamics during surgical procedures. Cases of profound hypotension after continuous labetalol infusions have been reported; however, there is limited evidence regarding the safety of intraoperative labetalol boluses. This audit examined the frequency of postoperative hypotension and bradycardia in 292 adult non-cardiac surgery patients treated with intraoperative labetalol boluses. Blood pressure and heart rate data were collected from the post-anaesthesia care unit and on the floor units for 24 hours after surgery. The median total intraoperative labetalol dose was 10mg. A total of 30/292 patients had *all-cause* postoperative hypotension within 24 hours of surgery, 26 of which had other medical or surgical precipitants. Fifteen patients developed bradycardia. There were no deaths or intensive care unit admissions attributed to labetalol. This audit demonstrates a low risk of *all-cause* postoperative hypotension (10%) and bradycardia (5%) after the use of small IV doses of intraoperative labetalol.

## Introduction

During surgery, patient anxiety, surgical stimulus and pain can all cause sympathetic stimulation and place increased demands on the cardiovascular system with the potential to precipitate major adverse cardiac events including myocardial ischaemia, infarction, arrhythmia and stroke (Reich et al 2002, Röhrig et al 2004). Labetalol is an antihypertensive agent with alpha and beta receptor blocking activity that can be administered orally, or intravenously as boluses or infusions. In conjunction with anaesthetic agents and analgesics, intravenous (IV) boluses of labetalol are commonly used by anaesthesiologists to help optimise patients’ blood pressure and reduce cardiovascular risks in the perioperative setting. An unpublished internal quality audit at our institution demonstrated intraoperative labetalol use in 4% of all non-cardiac surgical cases between 2010 and 2016.

Case reports in the literature have described episodes of severe and refractory hypotension which required rescue vasopressor and glucagon infusions after use of continuous IV labetalol infusions (Fahed et al 2008, Jivraj et al 2006). Based on these reports, our institutional policy mandated a prolonged period of labour-intensive haemodynamic monitoring in both the post-anaesthesia care unit (PACU) and on the floor for all patients who received any IV labetalol, regardless of the dose or dosing method (IV bolus or infusion). This new policy required postoperative blood pressure measurements every five minutes for 30 minutes, then every 15 minutes for one hour, then every 30 minutes for two hours, then every hour for six hours. Patients were also required to remain supine for three hours to avoid orthostatic hypotension. The new requirement for additional haemodynamic monitoring negatively impacted surgical patient flow and resulted in prolonged patient stays in the PACU, refusal to accept patient transfer to a regular floor bed, increased workload for nurses on the floor units and extra demand for intermediate care beds (IMCU).

The local institutional policy modification was solely based on adverse events reported from case reports after IV labetalol infusions, as there was limited evidence in the literature regarding the safety of small boluses of IV labetalol in the perioperative setting. As a result of this change, our anaesthesia quality improvement and patient safety group sought to determine whether the severity of postoperative hypotension and adverse events after intraoperative labetalol administration warranted these additional monitoring requirements. The specific purpose of this retrospective audit was to quantify the rates of postoperative hypotension, bradycardia and adverse events within a 24-hour period (Hecht et al 2019, Malsch et al 1991) after receiving small boluses of IV labetalol in the operating room and to help delineate its safety profile for postoperative monitoring requirements.

## Methods

The (Nova Scotia Health Authority Research Ethics Board) Research Ethics Board waived the need for research ethics board approval (5 February 2018) for this retrospective quality improvement audit under article 2.5 of the Tri-Council Policy Statement: Ethical Conduct for Research Involving Humans (2018).

A convenience sample of 310 consecutive surgical patients who received intraoperative IV labetalol boluses at an adult academic teaching hospital between 1 January and 30 June 2017 was identified from an electronic anaesthesia intraoperative monitoring system (Innovian, Drager Inc, Telford, PA) for retrospective analysis of 24 hour postoperative haemodynamic stability. The cohort included adult non-cardiac surgical patients who received IV boluses of labetalol in the operating room and were admitted to hospital for greater than 24 hours after their surgery. Patients undergoing outpatient procedures, those who received labetalol infusions and those admitted directly to the intensive care unit after surgery were excluded from analysis.

After identification of the cohort from the anaesthesia database, the electronic medical records for each patient were reviewed for a history of hypertension, type of hypertension medications prescribed and which hypertension medications were taken or held on the day of surgery. The patients’ age, sex and non-invasive blood pressure (NIBP) in the preoperative area were also recorded. Blood pressure measurements in the operating room were based on recordings taken with standard non-invasive blood pressure cuffs or arterial line readings at the time of the labetalol administration. The surgical procedure, type of anaesthetic, intraoperative blood pressure, heart rate (HR) and IV labetalol doses were extracted from the electronic intraoperative record. Postoperative records were then manually reviewed for heart rate, systolic blood pressure (SBP) and diastolic blood pressure (DBP) measurements in the 24-hour period after surgery. The blood pressure readings on the ward were based on NIBP readings taken by nursing staff at intervals prescribed as per ‘routine vital signs’ monitoring. The mean arterial pressure (MAP) was calculated (Gregory et al 2021) using the systolic and diastolic blood pressures (MAP = (SBP − DBP)/3 + DBP), charted by floor nursing staff. Prior to the new institutional policy for postoperative monitoring after labetalol administration, the standard vital signs assessment interval on the floor units was every four hours.

The primary outcome measures of this audit were the rates of hypotension and bradycardia in the 24-hour period after surgery. Hypotension was defined as a SBP less than 90mmHg or MAP less than 65mmHg (Liem et al 2020, Sessler et al 2018, Turan et al 2019) and bradycardia was defined as a heart rate less than 50 beats per minute (Röhrig et al 2004). Secondary outcomes were assessed in the cohort of patients who developed hypotension or bradycardia. These patients’ charts were reviewed for potential medical or surgical causes of the hypotension or bradycardia, need for admission to intermediate or critical care beds, or any other adverse events attributable to labetalol administration.

Statistical data were reported as counts and percentages, mean and standard deviation or median and interquartile range [IQR] where appropriate. The preoperative, intraoperative and PACU blood pressure and heart rate recordings were compared using ANOVA and Tukey’s test for multiple comparisons (Sigma Stat 12, Systat Software Inc, San Jose, CA). Proportion data was compared using Chi-square analysis. The level of statistical significance was set at p < 0.05.

## Results

A total of 310 consecutive patients who received intraoperative IV labetalol within the six-month study period were identified from the electronic anaesthetic records. Eighteen patients did not meet the inclusion criteria and were excluded from the analysis. The reasons for exclusion were as follows: direct postoperative ICU admissions (ten cases), incomplete charts or missing vitals (five cases), same day discharge (one case), case outside date criteria (one case) and cancellation due to hypertensive crisis before induction of anaesthesia (one case). Demographic data for the 292 patients meeting the inclusion criteria is shown in Table 1.

**Table 1:** Demographic data for all patients who received intraoperative IV labetalol (*n* = 292)

Item description	Count (%) or mean (*SD*)
Male/Female *n* (%)	141/151 (48%/52%)
Age (mean ± *SD*)	56 ± 20 years
Diagnosis of hypertension, *n* (%)	164 (56%)
Rx for home antihypertension medications, *n* (%)	154 (53%)
Surgical procedure category, *n* (%)	
Oral maxillofacial	71 (24%)
General	57 (20%)
Orthopaedics	60 (21%)
Vascular	29 (10%)
Otolaryngology	28 (10%)
Urology	22 (8%)
Gynaecology	8 (3%)
Neurosurgery	7 (2%)
Thoracic	6 (2%)
Ophthalmology	4 (1%)

*SD*: standard deviation; Rx: prescription.

Over half of the study cohort had a pre-existing history of hypertension. The majority of these hypertensive patients were prescribed one or two antihypertensive agents (Table 2). In accordance with Canadian Cardiology Society Guidelines (Duceppe et al 2017), angiotensin converting enzyme inhibitor (ACE-I) or angiotensin II receptor blocker (ARB) agents were typically withheld on the day of surgery (91%–94%, Table 2), while most patients were continued on their calcium channel or beta-blocker agents (70%–78%). General anaesthesia 266/292 (91%) was the most common anaesthetic type.

**Table 2:** Home antihypertensive agent type, including the proportion of patients taking that agent on day of surgery

Number of home antihypertensive agents per patient	Count n/*N* (%)	
No agents prescribed	138/292 (47%)	
One antihypertensive agent	64/292 (22%)	
Two antihypertensive agents	63/292 (22%)	
Three antihypertensive agents	23/292 (8%)	
Four antihypertensive agents	4/292 (1%)	
Home antihypertensive agent type	Number of patients on prescribed agent (n)	Number of patients taking agent on the day of surgery (n, %)
ACE-I	70	6/70 (9%)
ARB	34	2/34 (6%)
Beta blocker	49	38/49 (78%)
Calcium channel blocker	40	28/40 (70%)
Diuretic	70	22/70 (31%)
Alpha blocker	1	1/1 (100%)
Alpha/beta blocker combination	3	2/3 (67%)
Alpha agonist	4	2/4 (50%)
Nitrate	4	3/4 (75%)

ACE-I: angiotensin converting enzyme inhibitor; ARB: angiotensin II receptor blocker.

The median total intraoperative IV labetalol dose was 10mg (IQR 5–15 mg) and the maximum total dose given to one patient was 70mg. Statistically significant differences in MAP and HR were seen from the time of intraoperative labetalol administration and in the PACU after surgery (Table 3). The median number of HR and BP vital signs recorded during the patients’ stay in the PACU was eight readings (IQR 6–11), and for the floor units the median number of vital signs recorded over the full 24-hour postoperative period was six readings (IQR 5–7).

**Table 3:** Mean blood pressure and heart rate (±1 standard deviation)

Time of haemodynamic vital signs measurement	Systolic pressure, mmHg	Diastolic pressure, mmHg	MAP, mmHg	Heart rate, beats/minute
Preoperative	137 (21)	78 (12)	98 (13)	N/A
Intraoperative	171 (36)	87 (21)	115 (25)^a^	89 (22)^b^
PACU	149 (27)	79 (14)	102 (17)^a^	80 (14)^b^

MAP: mean arterial pressure; PACU: post-anaesthesia care unit.

Intraoperative represents the blood pressure at the time of the first labetalol dose administered in the operating room. The post-anaesthesia care unit (PACU) measurements were the set first recorded on arrival from the operating room. N/A – Preoperative heart rate was not recorded during data collection for this study.

a MAP–Tukey test, Preoperative versus Intraoperative p < 0.001, Intraoperative versus PACU p < 0.001, Preoperative versus PACU p = 0.03.

b Heart rate – ANOVA, Intraoperative versus PACU p < 0.001.

The rates of postoperative hypertension, hypotension and bradycardia events are shown in Table 4, along with the breakdown of where the events occurred. In the PACU, a higher proportion of patients were found to have ongoing hypertension requiring further treatment than those who developed hypotension in PACU (Chi-square p < 0.001). The lowest heart rates recorded in this study were 38 beats per minute while in the PACU, and 35 beats per minute while on a floor unit.

**Table 4:** Rates and occurrence locations of hypotension and bradycardia events for all patients given intraoperative labetalol (*n* = 292)

Event description	Number (%)
* All-cause* hypotension within 24 hours	30/292 (10%)
* All-cause* hypotension occurring in PACU^a^	16/292 (5%)
* All-cause* hypotension occurring on floor	10/292 (3%)
* All-cause* hypotension occurring in both PACU and the floor units	4/292 (1%)
Bradycardia within 24 hours	15/292 (5%)
Bradycardia in PACU requiring treatment with glycopyrrolate	3/292 (1%)
Ongoing hypertension in PACU requiring further treatment^a^	62/292 (21%)
Additional IV labetalol in PACU	31/292 (11%)
IV hydralazine in PACU	22/292 (8%)
Home antihypertensive administered in PACU	9/292 (3%)
Hypotension in PACU requiring treatment (phenylephrine *n* = 1, ephedrine *n* = 7)	8/292 (3%)

PACU: post-anaesthesia care unit.

a PACU hypotension versus PACU hypertension, p < 0.0001 Chi-square test.

Manual review of the narrative content in charts from the 30 patients with hypotension identified only four cases of postoperative hypotension (4/292 or 1.4%) without other contributing causes such as surgical complications, bleeding, residual anaesthetic, or cardiac events (Table 5). There were no documented episodes of complete heart block or asystole in the study cohort and there were no deaths or intensive care unit admissions directly attributed to labetalol.

**Table 5:** Details from manual chart review of the cohort (30/292) with documented postoperative hypotension

Details of associated events in the postoperative hypotension group (total *n* = 30)	Number (n)	Comments
Unexplained hypotension – no specific medical or surgical events identified	4	No causes or triggering events identified from manual review of patient records
Orthognathic corrective surgery	5	Induced hypotension used intraoperatively to minimise bleeding in the surgical field
Carotid endarterectomy	5	Altered haemodynamics are a common postoperative side effect with surgical manipulation of the carotid bulb and baroreceptors
Single hypotension reading with first blood pressure on PACU arrival	4	Possible residual anaesthetic agents still in effect at time of first blood pressure reading
Postoperative bleeding	3	Hypovolemia, all three patients required postoperative blood transfusions
Atrial fibrillation	2	ACS and suspected PE in one case, intraoperative cardioversion required in the other case, patient also took ARB that day
Low diastolic pressure in setting of recent opioid analgesic in PACU	3	Analgesic medication related while under observation in PACU
Sepsis	1	Postoperative cardiac arrest secondary to sepsis and hypoglycemia
Acute coronary syndrome	1	Postoperative cardiology management required for elevated troponin assay
Aspiration	1	Aspiration with airway management, postoperative death
Intraoperative hypotension	1	Patient required intraoperative phenylephrine infusion, then required labetalol for emergence hypertension

PACU: post-anaesthesia care unit; ACS: acute coronary syndrome; PE: pulmonary embolus; ARB: angiotensin II receptor blocker.

## Discussion

This retrospective audit demonstrated a low rate of *all-cause* postoperative hypotension (10%) for patients who received small doses of IV labetalol in the perioperative non-cardiac surgery setting. Moreover, the rate of postoperative hypotension without any associated medical or surgical causes that could be attributable to labetalol was only 1.4%. Importantly, there were no major adverse events such as death, heart block or ICU admissions directly attributed to labetalol administration in our cohort. Small intraoperative labetalol doses (median dose 10mg) demonstrated clinical and statistically significant reductions in both the MAP (11%) and HR (10%) from the time of administration in the operating room to arrival in the PACU.

Since our study was retrospective in design and lacked a control group, we chose to include *all-cause* hypotension as an outcome measure to allow for comparison with other postoperative hypotension studies in the literature. The 10% rate of *all-cause* postoperative hypotension found in this audit is lower than previous studies which have reported *all-cause* postoperative hypotension rates of 18% after abdominal surgery (Turan et al 2019) and 19% after various non-cardiac surgeries (Liem et al 2020). Neither of these previous studies on the rates of postoperative hypotension were directed specifically at the perioperative use of anti-hypertensive agents. In addition, only 3% (10/292) of our cohort had *all-cause* hypotension identified after transfer to the floor. Our audit findings would suggest that the use of small intraoperative labetalol doses did not result in excessive rates of postoperative hypotension events in comparison to the normal baseline rate of postoperative hypotension reported in the literature. Our local institutional policy requiring extensive postoperative haemodynamic monitoring for all cases where intraoperative labetalol was administered is unfounded based on the results of this audit.

Five of the thirty identified *all-cause* hypotension cases had only marginal hypotension with a MAP of 63–64mmHg. Although this met the study definition of hypotension, clinically this blood pressure level would not necessarily require medical intervention. Four of the *all-cause* hypotension cases occurred 10–15 hours after labetalol administration, which is outside the effective half-life of the medication (Malsch et al 1991, Peacock et al 2012, Varon & Marik 2008), making it unlikely that labetalol was the precipitant.

Two specific surgical groups exhibited the highest rates of postoperative hypotension. Five of the postoperative hypotension cases occurred in young healthy adults undergoing corrective orthognathic surgery, where the surgeons request intraoperative controlled hypotension to reduce bleeding and improve visualisation of the surgical field (Precious et al 1996). For these orthognathic cases the MAP is purposefully lowered to approximately 60mmHg during the surgery and allowed to normalise after cessation of the procedure. Mild postoperative hypotension in this young and otherwise healthy cohort may not necessarily be of clinical significance or warrant concern. Another five cases of postoperative hypotension were identified in the setting of carotid endarterectomy surgery, where surgical manipulation of the carotid can affect the carotid bulb and result in postoperative baroreceptor dysregulation. However, control of hypertension in carotid surgery patients is critical to reduce the risk of bleeding and hematoma formation which can lead to life-threatening airway compromise (Geniton 1990). After the surgical correction of carotid stenoses, it could be argued that aggressive blood pressure treatment may be preferable to uncontrolled hypertension to avoid potential postoperative hematoma formation.

In the present audit, a large proportion of the hypotension episodes (16/30 cases) occurred early in the postoperative period within the PACU setting, where all patients undergo close haemodynamic monitoring until they meet discharge criteria. Moreover, only 11 of these patients required some form of haemodynamic rescue medication in PACU for hypotension or bradycardia before discharge to the floor. In contrast, 53 of 292 study patients had ongoing *hypertension* in PACU requiring additional boluses of IV labetalol or hydralazine, and a further nine patients required administration of their home oral antihypertensive medications in the PACU. The standard practice of withholding ACE-I or ARB medications on the day of surgery in accordance with the current guidelines for non-cardiac surgery (Duceppe et al 2017) may have contributed to intraoperative hypertension and the need for both intraoperative labetalol administration and further postoperative antihypertensive treatments. In our experience, titrated doses of labetalol can be very effective for adjusting blood pressure in these patients.

Labetalol is a medication commonly used for ‘as-needed’ blood pressure control of hospitalised patients (Gaynor et al 2018, Lipari et al 2016, Weder & Erickson 2010) or treatment of hypertensive crisis (Hecht et al 2019, Peacock et al 2012) and labetalol has previously been recommended for management of perioperative hypertension (Fontes et al 2012, Haas & LeBlanc 2004, Lien & Bisognano 2012, Varon & Marik 2008). In contrast, IV hydralazine which has a delayed maximal effect is difficult to titrate and is generally not recommended for postoperative use (Fontes et al 2012, Haas & LeBlanc 2004, Lien & Bisognano 2012, Varon & Marik 2008). Interestingly, our local institutional policy does not require additional postoperative haemodynamic monitoring after intraoperative hydralazine administration, anecdotally this may have led to preferential usage of this sub-optimal medication in the perioperative setting.

There has been limited evidence published regarding the safety and efficacy of small IV labetalol boluses in the perioperative setting. Our study is the largest cohort of patients followed for a full 24 hours after small intraoperative doses of IV labetalol. Previous studies which were limited by smaller study cohorts or shorter observation periods have demonstrated the safety and efficacy of labetalol use in the perioperative setting. Singh et al (1992) conducted an open randomised trial comparing esmolol and small doses of IV labetalol in 22 geriatric patients undergoing ambulatory cataract surgery under local anaesthesia. They found that labetalol doses of 10–15mg safely lowered blood pressure without a significant decrease in heart rate. They also reported that their patients were discharged home three hours after surgery without any episodes of dizziness, or orthostatic hypotension. Another small study by Malsch et al (1991) reported no postoperative heart rate changes or hypotension for 24 hours in 30 patients who were given incremental IV labetalol doses of 20–140mg. Similarly, another study of 15 patients who were given IV labetalol in the recovery room reported ‘no untoward side effects’ and ‘adequate blood pressure control’ for four hours after treatment (Leslie et al 1987). A comparison study between IV labetalol boluses and sodium nitroprusside infusions for controlling blood pressure during carotid endarterectomy surgery (Geniton 1990) concluded that labetalol was efficacious in this setting and avoided the potential issues of tachyphylaxis, metabolic toxicity and myocardial ischaemia associated with sodium nitroprusside.

Not unexpectedly, two studies that used large doses of labetalol (170–4465mg) in the critical care setting for management of hypertensive crises demonstrated considerably higher hypotension rates (18-30%) than our study (Hecht et al 2019, Wesselink et al 2018). However, these studies are not directly comparable to our study as they did not occur in the perioperative setting and the labetalol infusions were administered over a longer treatment period (8–24 hours).

This retrospective audit is limited due to the lack of a control group, and we were unable to directly attribute cause and effect between hypertension treatment and postoperative hypotension. The cause of postoperative hypotension is often multifactorial. In this retrospective audit, we were unable to fully isolate labetalol from other medical events as the sole cause of hypotension, as such we also chose to report *all-cause* hypotension to demonstrate a low incidence of postoperative hypotension in our cohort, regardless of etiology. Our results apply to non-cardiac surgical patients who were treated with small boluses of IV labetalol, well below the maximum recommended dose of 300 mg (Haas & LeBlanc 2004). The audit also was limited in that the typical time between routine vital signs assessment on the floor unit was every four hours, and episodes of hypotension within those four-hour intervals would not be captured without continuous monitoring (Turan et al 2019). Ideally, all postoperative patients would have continuous haemodynamic monitoring, but at present the costs and infrastructure requirements are prohibitive and a more rational risk-based approach to monitoring is required.

## Conclusion

In summary, our audit demonstrates a low risk of postoperative hypotension in patients given small intraoperative boluses of labetalol during non-cardiac surgeries. The rate of *all-cause* postoperative hypotension in our labetalol cohort is below the published baseline rates of *all-cause* postoperative hypotension for non-cardiac surgery patients. These results will assist in rationalising institutional postoperative monitoring requirements for surgical patients given small doses of intravenous labetalol.

## Key points

1. The rates of postoperative hypotension and bradycardia were low after small intraoperative doses of labetalol
2. No major adverse events (death, heart block, intensive care unit admission) were attributed to labetalol administration
3. Administration of small doses of intraoperative labetalol does not necessitate intensive postoperative hemodynamic monitoring
